# Spontaneous Renal Allograft Rupture Caused by Acute Tubular Necrosis: A Case Report and Review of the Literature

**DOI:** 10.1155/2017/9158237

**Published:** 2017-07-20

**Authors:** Deepak Shankar Ray, Sharmila Thukral

**Affiliations:** Rabindranath Tagore International Institute of Cardiac Sciences, Narayana Health Hospitals, 124 EM Bypass, Mukundapur, Kolkata, India

## Abstract

Renal allograft rupture (RAR) is a rare but lethal complication of renal transplantation. It potentially threatens graft and patient survival. RAR is frequently associated with acute rejection, but other causes like renal vein thrombosis and acute tubular necrosis have also been observed. Most commonly a graft nephrectomy is required, but graft repair can also be attempted in selected cases to salvage the graft. Herein, we describe a rare case of spontaneous renal allograft rupture in the early posttransplant period due to acute tubular necrosis. A 42-year-old male, living donor renal allograft recipient, experienced RAR on the sixth posttransplant day. Surgical exploration showed two lacerations of 10 cm and 5 cm length at the upper and mid pole of the kidney. Histologically, the graft demonstrated acute tubular injury; no features of humoral or cellular rejection were identified. The successful management of this complication resulted in the salvage of the patient and the graft. This case demonstrates that early diagnosis and prompt treatment of a life-threatening RAR can salvage the graft.

## 1. Introduction

Spontaneous renal allograft rupture is a rare yet potentially life-threatening complication of renal transplantation. It is associated with a high incidence of graft loss [1]

The consequences are fatal in 6% of the cases and graft loss is the outcome in another 53% [2]

Most of these cases are immunologically mediated and caused by acute rejection. Acute tubular necrosis is only rarely responsible for this complication [3, 4].

With improvements in the immunosuppressive regimens, the incidence of this complication has decreased. Recognition and prompt management of allograft rupture are important because of its likely devastating outcomes. Usually, graft nephrectomy is necessary in these cases, but conservative surgical intervention has also been tried successfully.

We report an unusual case of a renal allograft recipient who developed a spontaneous RAR. The rupture was secondary to a relatively uncommon cause, acute tubular necrosis. The graft was successfully salvaged by surgical repair.

This case emphasises that the transplant team must be aware of this fatal complication and the current management strategies.

## 2. Case History

A 42-year-old gentleman, suffering from End-Stage Renal Disease (ESRD) secondary to vesicoureteral reflux (VUR). VUR was detected at the age of four years. He developed progressive proteinuria 12 years later followed by slowly progressive decline of renal function culminating in ESRD and was started on hemodialysis in Jan 2010. He underwent a live spousal blood group compatible renal transplant on 19th Feb 2010. The graft was lost to chronic allograft nephropathy in 2015 and second live donor renal transplant was planned. Donor was 33-year-old male, altruistic with HLA mismatch 3/6, ABO compatible, and CDC cross-match negative. Optical internal urethrotomy was done one day prior to transplant as he had urethral stricture diagnosed by micturating cystourethrogram. Induction was done with antithymocyte globulin in the dose of 3 mg/kg and triple immunosuppression was given with tacrolimus, mycophenolate mofetil, and prednisolone. The second transplant was done on 20th April 2016. Donor nephrectomy was done laparoscopically, the warm ischaemia time being 10 minutes. There was no injury to the renal vein or artery during donor nephrectomy. After anastomosis of the renal vein to the external iliac vein, there was avulsion at the venous anastomotic site. There was no thrombosis or stenosis of the vein and no heparin was required intraoperatively.

Venous avulsion was repaired with 6.0 Prolene immediately, after which the arterial and venous blood flows were normal. The recipient was hemodynamically stable in the intraoperative and immediate posttransplant period. Immediate postoperative graft function was poor.

In the next six postoperative days, his urine output remained around 2–4 liters/day. His serum creatinine came down very slowly from 9.97 mg/dL to 8.37 mg/dL by the fifth posttransplant day. Drain fluid decreased from 190 ml/day to 80 ml/day by the fifth posttransplant day. Hemoglobin remained stable, and no significant perigraft collection was seen on sonography. In view of poor allograft function, a renal biopsy was planned on sixth postoperative day, but his drain volume suddenly increased on that day and a frank hemorrhagic collection of 1 liter was drained within half an hour. The patient complained of severe abdominal pain and the urine output dropped. The graft became swollen and tender.

**Figure 1:**
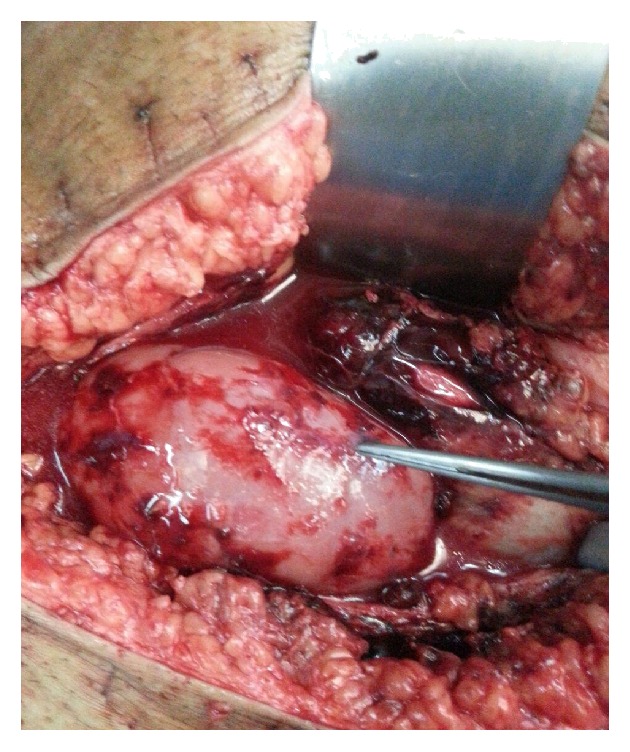
Two ruptures, 10 cm and 5 cm one at upper pole and one at midportion, respectively.

Aggressive resuscitation was started and the patient was immediately reexplored in the operation theatre. A hematoma with large amount of blood clots was evacuated. There were two ruptures in the transplant kidney, 10 cm and 5 cm, one at upper pole and one at midportion, respectively, along the convex border (Figure 1). There was no leak at the anastomotic site and no other source of bleeding was found. The coagulation profile of the patient was normal. Ruptures were repaired with 3–0 Prolene hemolock and reinforced by TACOSIL.

An allograft biopsy was done in the operating room.

The patient was shifted to transplant unit and the patient was given two sessions of plasmapheresis followed by 5 gm of IVIG on the first and second posttransplant day, while awaiting the graft biopsy report.

The donor specific antibodies were negative by flow cytometry. Histopathology showed normocellular and uniform glomeruli. There were no capillary wall thickening and no fibrin thrombi or necrosis. Tubules showed vacuolation and degeneration and necrosis of epithelial cells. There was severe acute tubular necrosis. There was no tubulitis. There were no interstitial infiltration, fibrosis, and endarteritis. C4d stain was negative in the peritubular capillaries and glomerular capillaries.

**Figure 2:**
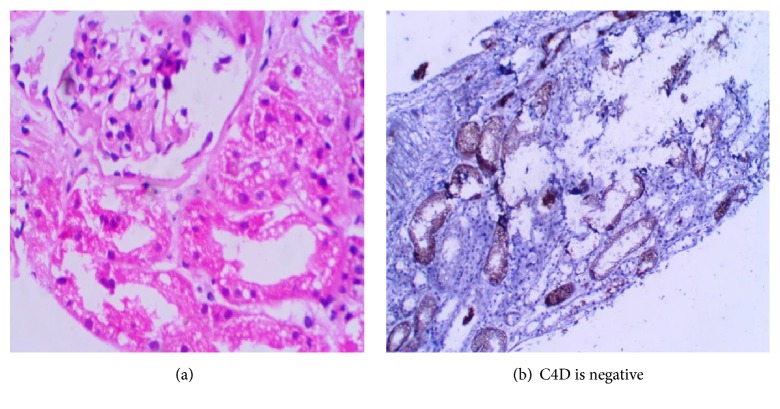


In Figures 2(a) and 2(b) the patient improved symptomatically; drain volume came down to less than 50 ml by the sixteenth posttransplant day. It also became mainly serous collection and the drain was removed after 8 days.

**Figure 3:**
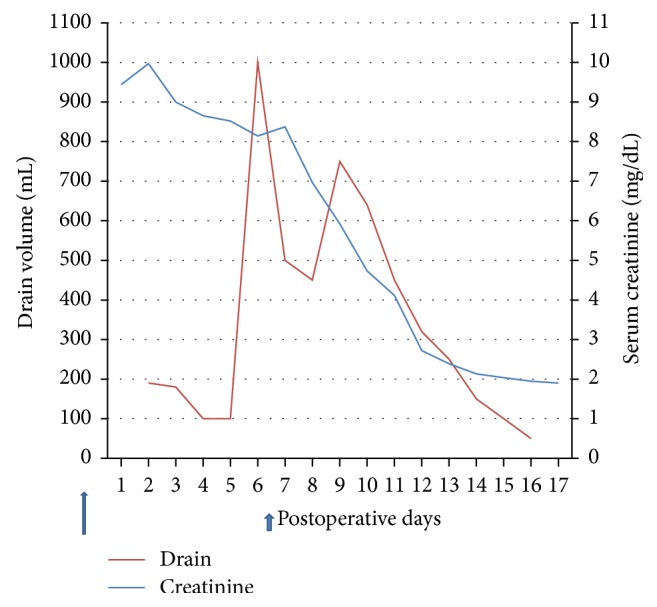
Time course of events.

His graft function improved significantly and he was discharged after 10 days of repair of the rupture with good urine output and a serum creatinine of 1.9 mg/dl (Figure 3).

## 3. Discussion

Renal allograft rupture is defined as a superficial or deep tear of the renal capsule as well as renal parenchyma [5].

It was first reported in 1968 by Murray et al. [6].

The prevalence of RAR ranges from 0.3% to 9.6% with a mean of 3.4% [7].

It typically occurs within three weeks after transplantation [4].

The most frequent cause of RAR is acute graft rejection. It is the main predisposing factor in 60–80% of the cases [8].

Other factors contributing to RAR include ischaemic acute kidney injury, acute tubular necrosis, damaged hilar lymphatic channels, renal vein thrombosis, ureteral obstruction with subsequent hydronephrosis, renal biopsy, trauma, nephrostomy tubes, and development of renal cell cancer [4, 5, 9–11].

The pathogenetic mechanism of RAR is not fully understood. Cortical and capsular ischaemia resulting from interstitial oedema and cellular inflammatory cell infiltration, in the setting of acute rejection, is considered the major cause of RAR by exerting capsular tension, tearing, and rupture. This may occur even many years after transplantation [12].

Interstitial oedema was found in 85% cases and major ischaemic damage in 15%. Graft biopsy is the etiological factor in 15% and hydronephrosis in 4%.

Hemodialysis with heparin is the possible factor in 46% cases [13]. Clinically, RAR is most commonly characterised by sudden onset of abdominal pain, graft tenderness, and swelling, and decreased haematocrit with hemorrhagic shock. It is generally associated with oliguria or anuria, sometimes with gross hematuria, fever, and bleeding from incision. Clinical diagnosis may be confirmed by ultrasound or CT scan [7]. Immediate ultrasound evaluation can confirm the diagnosis with 87% sensitivity and 100% specificity [14].

The management of RAR is urgent exploratory operation to control the bleeding, perform nephrectomy if indicated, and evacuate the hematoma to reduce the possibility of secondary infection.

Early reports of conservative management of RAR showed poor results with less than 30% success rates. The high rate of graft nephrectomies was due to the failure to control bleeding and inability to reverse rejection, postoperative multiorgan failure, and uncontrollable coagulopathy. Over the past two decades, improvements in the surgical techniques have significantly reduced the transplant related mortality and morbidity. Current literature demonstrate that the ruptured grafts can be salvaged with a success rate as high as 80%. Recurrent rupture occurs in only 5% patients [15].

With salvage rates between 40 and 100% and variable long-term complications and performance of the repaired graft, it is worth trying to salvage spontaneously ruptured grafts. Only in those patients whose hemodynamic status cannot be stabilised by appropriate aggressive hemodynamic support, graft nephrectomy should be considered as a definite treatment [1, 8, 9].

## 4. Conclusion

RAR is still encountered in clinical practice. In spite of advances in immunosuppression, most of the cases are due to acute rejection. Acute tubular necrosis is an uncommon cause of RAR. The life-threatening nature of this complication mandates early recognition and treatment. Although transplant nephrectomy is a definite treatment, with advances in the surgical techniques, attempts can be made to salvage the graft when hemorrhage can be controlled and RAR does not compromise survival of the patient.

## References

[1] Gomez-Veiga F., Chantada-Abal V., Garcia-Buitron J., Gonzalez-Martin M. (1993). Spontaneous rupture of transplanted kidney. Experience with 500 transplants. *Arch Esp Urol*.

[2] Van Der Viliet J. A., Kootstra G., Tegzess A. M. (1980). Managenent of rupture in allograft kidney. *The Netherlands Journal of Surgery*.

[3] Hochleitner B. W., Kafka R., Spechtenhauser B. (2001). Renal allograft rupture is associated with rejection or acute tubular necrosis, but not with renal vein thrombosis. *Nephrology Dialysis Transplantation*.

[4] Szenohradszky P., Smehák G., Szederkényi E. (1999). Renal allograft rupture: a clinicopathologic study of 37 nephrectomy cases in a series of 628 consecutive renal transplants. *Transplantation Proceedings*.

[5] Shahrokh H., Rasouli H., Zargar M. A., Karimi K., Zargar K. (2005). Spontaneous kidney allograft rupture. *Transplantation Proceedings*.

[6] Murray J. E., Wilson R. E., Tilney N. L. (1968). Five years' experience in renal transplantation with immunosuppressive drugs: survival, function, complications, and the role of lymphocyte depletion by thoracic duct fistula. *Annals of Surgery*.

[7] Azar G. J., Zarifian A. A., Frentz G. D., Jesi R. J., Ethredge E. E. (1995). Renal allogrsft rupture: a clinical review. *Clinical Transplantation*.

[8] Heimbach D., Miersch W. D., Buszello H., Schoeneich G., Klehr H. U. (1995). Is the transplant‐preserving management of renal allograft rupture justified?. *British Journal of Urology*.

[9] Garcia Sanchez de la Nieta M. D., Sánchez-Fructuoso A. I., Alcázar R. (2004). Higher graft salvage rate in renal allograft rupture associated with acute tubular necular. *Transplantation Proceedings*.

[10] Busi N., Capocasale E., Mazzoni M. P. (2004). Spontaneous renal allograft rupture without acute rejection. *Acta Bio-Medica*.

[11] Fernandez-Juarez G., Pascual J., Burgos F. J. (1998). Late rupture of the renal graft: not always graft rejection. *Nephrology Dialysis Transplantation*.

[12] Askandarani S., Aloudah N., Al Enazi H., Alsaad K. O., Altamimi A. (2011). Late renal allograft rupture associated with cessation of immunosuppression following graft failure. *Case Reports in Transplantation*.

[13] Goldman M., De Pauw L., Kinnaert P., Vereerstraeten P., Van Geertruyden J., Toussaint C. (1981). Renal allograft rupture: possible causes and results of surgical conservative management. *Transplantation*.

[14] Oesterwitz H., Strobelt V., May G., Horlbeck R. (1990). Spontaneous kidney transplant rupture- effect of immunosuppressive therapy on incidence of rupture: a clinical review. *Z Urol Nephrol*.

[15] Homan W. P., Cheigh J. S., Kim S. J. (1977). Renal allograft fracture: clinicopathological study of 21 cases. *Annals of Surgery*.

